# Supplementary material for: Network curvature as a hallmark of brain structural connectivity
Source: Nat Commun. 2019 Oct 30;10:4937. doi: 10.1038/s41467-019-12915-x (PMC6821808; doi:10.1038/s41467-019-12915-x)
Supplement: Supplementary file 1 — Supplementary Information [file 41467_2019_12915_MOESM1_ESM.pdf]

Supplementary Information
Network Curvature as a Hallmark of Brain Structural Connectivity

Hamza Farooq[1,*], Yongxin Chen[2], Tryphon T. Georgiou[3], Allen Tannenbaum[4], and Christophe Lenglet[5]

[1]Department of Electrical and Computer Engineering, University of Minnesota, MN
[2]School of Aerospace Engineering, Georgia Institute of Technology, GA
[3]Department of Mechanical and Aerospace Engineering, University of California, Irvine, CA
[4]Department of Computer Science and Applied Mathematics & Statistics, Stony Brook University, NY
[5]Center for Magnetic Resonance Research, University of Minnesota, MN
[*]faroo014@umn.edu

Supplementary Note 1

Nodes (cortical/ sub-cortical areas) with high curvature, strength and betweenness centrality from the high resolution (998 x 998) connectivity matrices from Hagmann et al. 2008[1]. The tables below list the top 25% of nodes appearing in 3 (or more) participants out of 5, with the highest curvature, strength and centrality. Cortical areas only found by curvature are highlighted in red.

Node Curvature<br>High resolution matrices (Hagmann et al. 2008)	
**Left Hemisphere**	**Right Hemisphere**
Lateral orbito-frontal	
Pars orbitalis	Pars orbitalis
Pars triangularis	Pars triangularis
Pars opercularis	Pars opercularis
Rostral middle-frontal	Rostral middle-frontal
Superior frontal	Superior frontal
Caudal middle-frontal	Caudal middle-frontal
Precentral	Precentral
	Isthmus cingulate
Rostral anterior cingulate	
Caudal anterior cingulate	
Posterior cingulate	
Postcentral	
Superior parietal	Superior parietal
Inferior parietal	Inferior parietal
Precuneus	Precuneus
Cuneus	
Pericalcarine	
Lateral occipital	Lateral occipital
	Fusiform
Lingual	
Inferior temporal	Inferior temporal
Superior temporal	Superior temporal
	Transverse temporal

Node Betweenness Centrality<br>High resolution matrices (Hagmann et al. 2008)	
**Left Hemisphere**	**Right Hemisphere**
Lateral orbito-frontal	Lateral orbito-frontal
Medial orbito-frontal	
Pars triangularis	Pars triangularis
Superior frontal	Superior frontal
Caudal middle-frontal	Caudal middle-frontal
Precentral	Precentral
Caudal anterior cingulate	Caudal anterior cingulate
Posterior cingulate	Posterior cingulate
Isthmus cingulate	
Postcentral	
Superior parietal	Superior parietal
Inferior parietal	
Precuneus	Precuneus
Cuneus	Cuneus
Pericalcarine	Pericalcarine
Lateral occipital	Lateral occipital
Superior temporal	
	Supra-marginal
	Inferior temporal
	Middle temporal
	Banks of sup. temporal sulcus

Node Strength<br>High resolution matrices (Hagmann et al 2008)	
**Left Hemisphere**	**Right Hemisphere**
Medial orbito-frontal	
Superior frontal	Superior frontal
Precentral	Precentral
	Paracentral
	Caudal anterior cingulate
Posterior cingulate	Posterior cingulate
Isthmus cingulate	Isthmus cingulate
Postcentral	Postcentral
Superior parietal	Superior parietal
Inferior parietal	Inferior parietal
Precuneus	Precuneus
Cuneus	Cuneus
Pericalcarine	Pericalcarine
Lateral occipital	Lateral occipital
Inferior temporal	
Middle temporal	Middle temporal
Banks of sup. temporal sulcus	Banks of sup. temporal sulcus
	Superior temporal
	Transverse temporal

Supplementary Note 2

Nodes (cortical/ sub-cortical areas) with high curvature, strength and betweenness centrality from the lower resolution (116 x 116) connectivity matrices generated from the MGH-USC HCP Consortium DSI datasets. The tables below list the top 25% of nodes appearing in 18 (or more) participants out of 33, with the highest curvature, strength and centrality. Cortical areas only found by curvature are highlighted in red.

Node Curvature<br>Lower resolution matrices (MGH-USC HCP)	
**Left Hemisphere**	**Right Hemisphere**
Precentral	Precentral
Superior frontal	Superior frontal
Middle frontal	Middle frontal
Pars opercularis	Pars opercularis
Pars triangularis	Pars triangularis
	Supplementary motor area
Medial frontal	Medial frontal
Heschl's gyrus	Heschl's gyrus
	Hippocampus
	Lingual
	Caudate
	Putamen
Pallidum	

Node Betweenness Centrality<br>Lower resolution matrices (MGH-USC HCP)	
**Left Hemisphere**	**Right Hemisphere**
Superior frontal	Superior frontal
Middle frontal	Middle frontal
Pars triangularis	Pars triangularis
Supplementary motor area	
Medial frontal	Medial frontal
	Fusiform
Cerebellum	Cerebellum

Node Strength<br>Lower resolution matrices (MGH-USC HCP)	
**Left Hemisphere**	**Right Hemisphere**
Precentral	Precentral
Superior frontal	Superior frontal
Middle frontal	Middle frontal
Pars triangularis	Pars triangularis
Supplementary motor area	Supplementary motor area
Medial frontal	Medial frontal
	Fusiform
Superior parietal	Superior parietal
Pallidum	Pallidum
Cerebellum	Cerebellum

Supplementary Note 3

Nodes (cortical areas), and corresponding functional communities, with significant differences in structural connectivity between age groups. Results shown below were obtained using the Gordon[2] atlas with 333 nodes, and provide raw p-values, significant after type I error correction using the Holm-Sidak method. Details regarding nodes (number etc.) of Gordon atlas[2] can be downloaded from `https://sites.wustl.edu/petersenschlaggarlab/parcels-19cwpgu/`[2].

Hemisphere	Node #	Community	Human Connectome Project LifeSpan Data			
			Curvature	Strength	Betweenness Centrality	Clustering Coefficient
Left	1	Default	2.40E-05			7.85E-09
	5	Visual			3.48E-05	
	13	Retrospleinal Temporal				2.13E-02
	15	Visual		9.64E-07	6.41E-03	
	21	Cingulo-Opercular				3.60E-02
	22	Cingulo-Opercular				1.94E-04
	27	Cingulo-Opercular				1.43E-02
	35	SM Hand		1.15E-05		
	73	None		3.66E-05	6.42E-04	
	80	Ventral Attention		7.36E-07		
	91	Dorsal Attention			1.33E-02	
	94	Default	7.05E-06		6.82E-03	
	124	None		1.14E-04		
	130	Retrospleinal Temporal				2.84E-03
	160	Auditory		7.22E-06		
Right	162	Default			1.02E-03	
	175	Visual			6.42E-04	
	185	Cingulo-Opercular				2.07E-04
	245	Cingulo-Opercular		5.86E-07		
	254	Cingulo-Parietal	2.37E-05			
	263	Visual	3.23E-06			
	287	None		1.18E-08		
	292	None		1.66E-07		
	303	None		1.41E-06		

Supplementary Note 4

Nodes (cortical areas), and corresponding functional communities, with significant differences in structural connectivity due to ASD. Results shown below were obtained using the Gordon[2] atlas with 333 nodes, and provide raw p-values, significant after type I error correction using the Holm-Sidak method. Details regarding nodes (number etc.) of Gordon atlas[2] can be downloaded from `https://sites.wustl.edu/petersenschlaggarlab/parcels-19cwpgu/`[2].

Hemisphere	Node #	Community	San Diego State University Data (SDSU)				Trinity Center (TC) Data			
			Curvature	Strength	Betweenness Centrality	Clustering Coefficient	Curvature	Strength	Betweenness Centrality	Clustering Coefficient
Left	1	Default		9.79E-12		3.40E-03				
	5	Visual			3.70E-03					
	8	Visual				4.10E-02				
	15	Visual		5.68E-07	1.34E-02		8.39E-05	2.30E-11		8.33E-06
	27	Cingulo-Opercular		1.28E-05						
	45	SM Hand			9.50E-03					
	58	SM Hand							3.06E-02	
	71	Cingulo-Opercular							2.50E-03	
	72	Cingulo-Opercular			2.00E-04					
	73	None							7.40E-03	
	80	Ventral Attention	4.48E-05					2.64E-11	4.05E-05	
	129	None					1.31E-05		4.27E-05	
	134	None			9.70E-03		1.93E-05	8.85E-06		
	138	Visual		1.60E-05						
	160	Auditory						1.00E-04		
Right	174	Retrospleinal Temporal								1.13E-02
	175	Visual	2.41E-08				5.99E-06			
	191	SM Hand						4.16E-05		
	275	Dorsal Attention	9.39E-07							
	292	None	4.79E-06							

Supplementary Note 5

This section describes the patterns of structural connectivity changes associated with ASD, as they relate to phenotypic measures. To carry out the analysis, correlations were computed between nodes with significant differences between ASD and TD (as identified by nodal measures i.e., curvature, strength, betweenness centrality and clustering coefficient) and affected phenotypic measures. Supplementary Figures 5 and 6 show significant correlations (in red color) with p-value less than 0.05. Following is a brief description of the information uniquely provided by node curvature in relation to phenotypic measures.

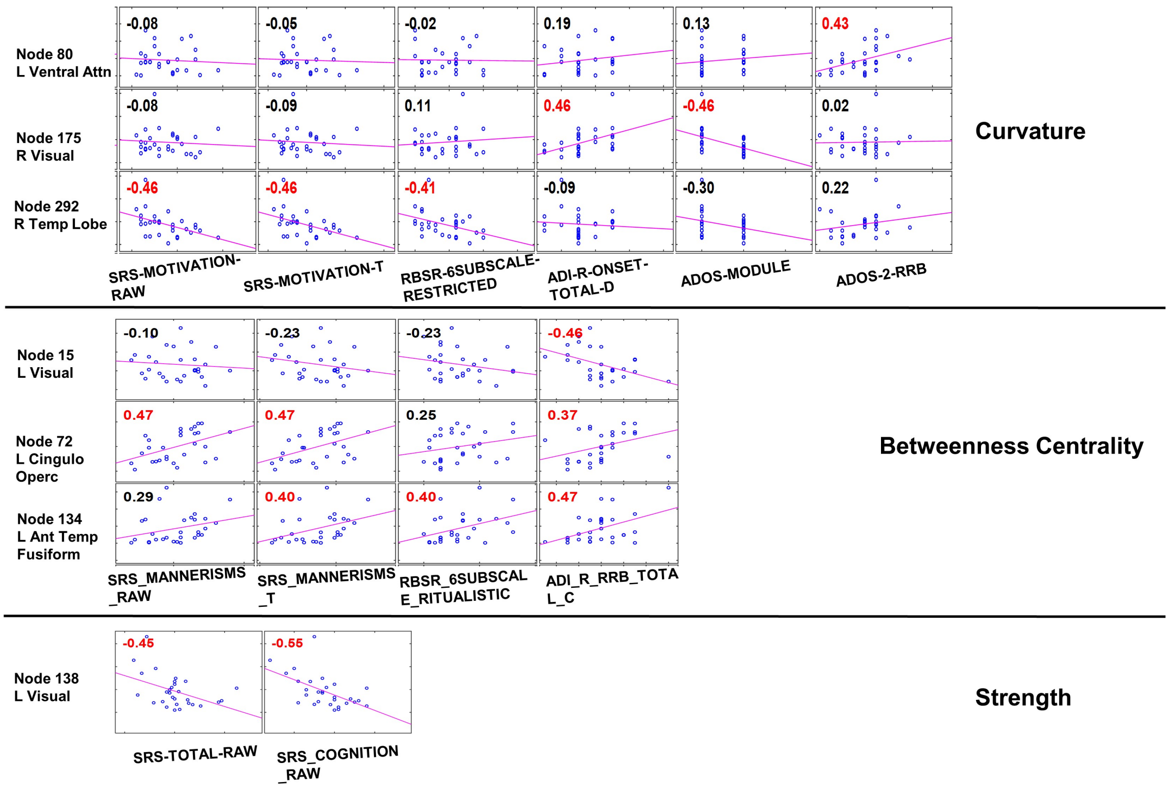

Supplementary Figure 5. Correlation between nodes with statistically significant differences in structural connectivity of ASD and TD subjects, and phenotypic measures with statistically significant differences between groups using San Diego State University (SDSU) data.

A recent study[3], based on resting-state functional MRI, reported that Social Responsiveness Scale[4] (SRS) sub-factors (social awareness, cognition, communication, motivation and autistic mannerisms) negatively correlated with the functional connectivity strength of the default modes network (DMN), consistent with prior studies, as discussed by the authors[3]. They also reported that levels of hyperactivity/impulsiveness and inattention behavioral problems were positively correlated with the functional connectivity strength of the executive control network (ECN). The cerebellum network had higher functional connectivity in ASD, compared to TD individuals. Finally, repetitive behavior has been reported to relate to the functional connectivity of the temporal lobes[5].

Here in this study, based on structural connectivity, node strength (of identified brain areas) shows similar behavior of negative correlation with SRS sub-factors (as shown in Supplementary Figures 5 and 6). However, not all sub-factors are identified by node strength or any other node measure. Curvature of the right temporal lobe (Brodmann area 38, which is involved in emotional and social processing) and SRS sub-factor Motivation (both Raw and converted T-scores), and Repetitive Behavior Scale-Revised (RBSR), are found to be negatively correlated. The left orbito-frontal cortex (ECN) curvature was also uniquely identified to correlate positively with the Autism Diagnostic Observation Schedule (ADOS-2) Restricted and Repetitive behavior scale. This is in line with the above mentioned studies and supplement the information provided by other node measures. Additionally, curvature of the anterior division of the temporal fusiform cortex (involved in recognition tasks, such as body and faces) positively correlated with several Child Behavior Checklist (CBCL) scores (e.g. Attention, Aggressive behavior) and RBSR sub-factor Self-injurious behavior. Finally, Supplementary Figure 6 shows that curvature of the left visual area negatively correlated with SRS sub-factor Mannerisms, RBSR sub-scale Restricted Interests and CBCL sub-factor Thoughts Problems.

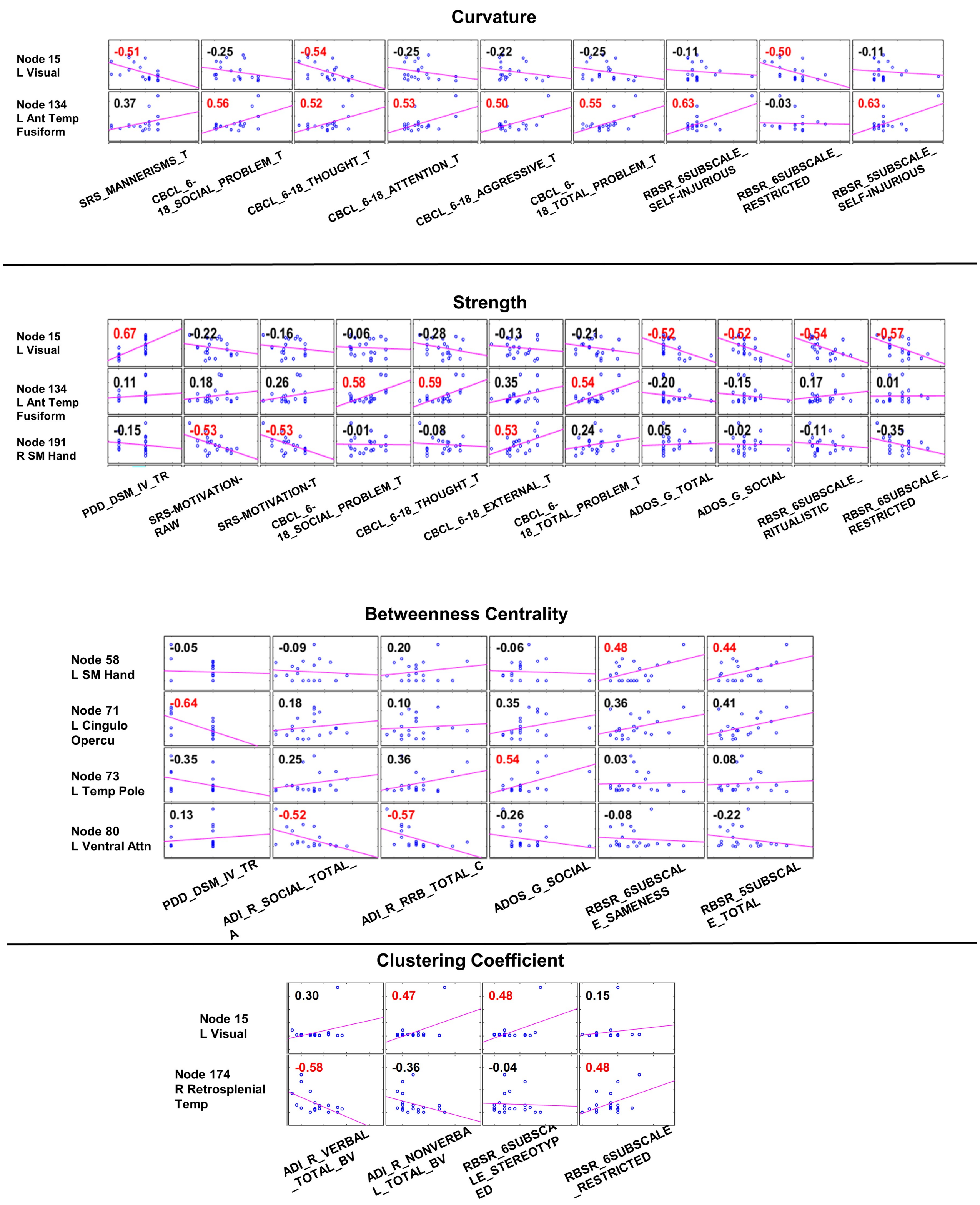

Supplementary Figure 6. Correlation between nodes with statistically significant differences in structural connectivity of ASD and TD subjects, and phenotypic measures with statistically significant differences between groups using Trinity Center for Health Sciences (TC) data.

Supplementary Note 6

Topological Entropy of a graph G is the logarithm of the spectral radius of the adjacency matrix A, i.e., the logarithm of the maximum of the absolute values of the eigenvalues of A[6].

Supplementary Note 7

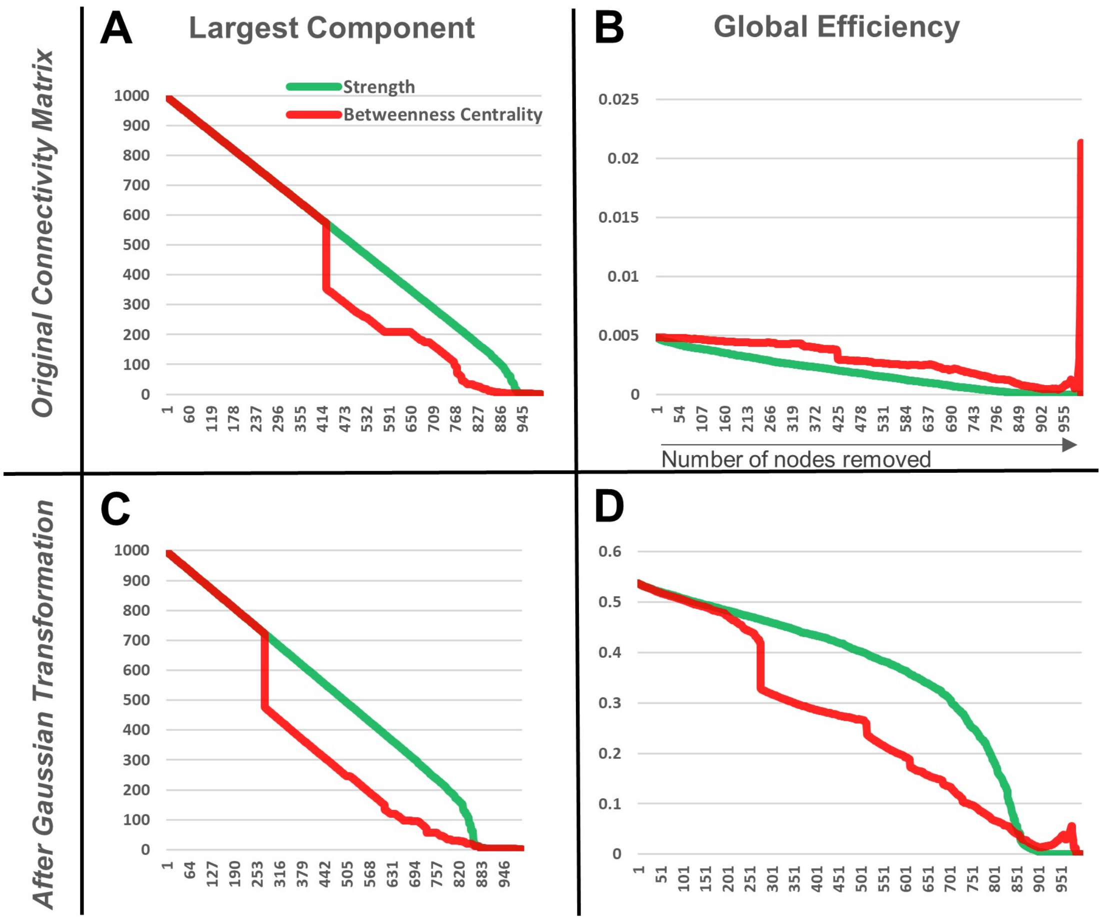

Supplementary Figure 7. Reproduction of robustness analysis presented in Alstott et al[7], using node deletion for the high resolution connectivity matrices (998×998) from Hagmann et al[1]. The size of the largest component and global efficiency are computed (with or without transformation of the connectivity matrix weights) after targeted removal of nodes with decreasing strength or betweenness centrality. The top row shows results for the original connectivity matrix while the bottom row shows results after Gaussian transformation of its weights. Note that results shown in panel C and D are similar to Fig. 3 in previous work[7].

Supplementary Note 8

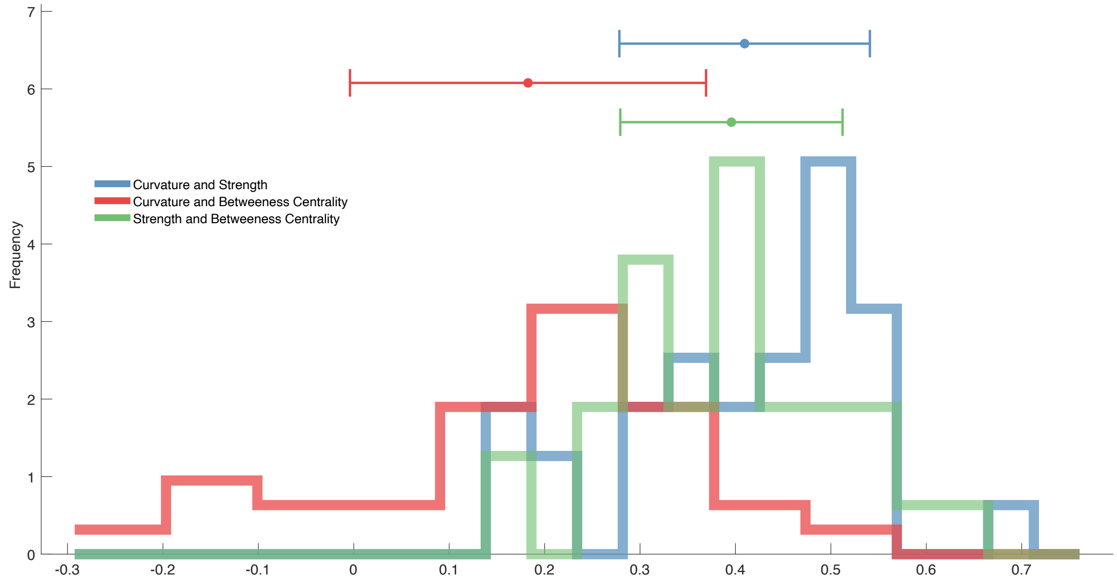

Supplementary Figure 8. Correlation between different node measures using the low-resolution MGH-USC HCP dataset (33 individuals). The histograms approximate the distribution of correlation (over the 33 datasets) between pairs of nodal measures computed at 116 nodes (AAL template).

References

1. Hagmann, P. *et al.* Mapping the structural core of human cerebral cortex. *PLOS Biol.* **6**, 1–15 (2008).
2. Gordon, E. M. *et al.* Generation and Evaluation of a Cortical Area Parcellation from Resting-State Correlations. *Cereb. Cortex* **26**, 288–303 (2014).
3. Chia Min, C., Pinchen, Y., Ming, W., Tzu, C. & Teng, H. Deriving and validating biomarkers associated with autism spectrum disorders from a large-scale resting-state database. *Sci. Reports* **9**, 9043 (2019).
4. Constantino, J. N. *et al.* Validation of a brief quantitative measure of autistic traits: Comparison of the social responsiveness scale with the autism diagnostic interview-revised. *J. Autism Dev. Disord.* **33**, 427–433 (2003).
5. Abbott, A. *et al.* Repetitive behaviors in autism are linked to imbalance of corticostriatal connectivity:a functional connectivity mri study. *Soc. cognitive affective neuroscience* **13** (2017).
6. Chen, Y., Georgiou, T., Pavon, M. & Tannenbaum, A. Robust transport over networks. *IEEE Transactions on Autom. Control.* **62**, 4675–4682 (2017).
7. Alstott, J., Breakspear, M., Hagmann, P., Cammoun, L. & Sporns, O. Modeling the impact of lesions in the human brain. *PLOS Comput. Biol.* **5**, 1–12 (2009).
