# Supplementary material for: Network curvature as a hallmark of brain structural connectivity
Source: Nat Commun. 2019 Oct 30;10:4937. doi: 10.1038/s41467-019-12915-x (PMC6821808; doi:10.1038/s41467-019-12915-x)
Supplement: Supplementary file 3 — Reporting Summary [file 41467_2019_12915_MOESM3_ESM.pdf]

nature research

Corresponding author(s): Hamza Farooq

Last updated by author(s): Sep 29, 2019

Reporting Summary

Nature Research wishes to improve the reproducibility of the work that we publish. This form provides structure for consistency and transparency in reporting. For further information on Nature Research policies, see Authors & Referees and the Editorial Policy Checklist.

Statistics

For all statistical analyses, confirm that the following items are present in the figure legend, table legend, main text, or Methods section.

n/a	Confirmed	
☐	☒	The exact sample size (n) for each experimental group/condition, given as a discrete number and unit of measurement
☒	☐	A statement on whether measurements were taken from distinct samples or whether the same sample was measured repeatedly
☐	☒	The statistical test(s) used AND whether they are one- or two-sided<br>*Only common tests should be described solely by name; describe more complex techniques in the Methods section.*
☒	☐	A description of all covariates tested
☐	☒	A description of any assumptions or corrections, such as tests of normality and adjustment for multiple comparisons
☒	☐	A full description of the statistical parameters including central tendency (e.g. means) or other basic estimates (e.g. regression coefficient) AND variation (e.g. standard deviation) or associated estimates of uncertainty (e.g. confidence intervals)
☐	☒	For null hypothesis testing, the test statistic (e.g. F, t, r) with confidence intervals, effect sizes, degrees of freedom and P value noted<br>*Give P values as exact values whenever suitable.*
☒	☐	For Bayesian analysis, information on the choice of priors and Markov chain Monte Carlo settings
☒	☐	For hierarchical and complex designs, identification of the appropriate level for tests and full reporting of outcomes
☒	☐	Estimates of effect sizes (e.g. Cohen's d, Pearson's r), indicating how they were calculated

Our web collection on statistics for biologists contains articles on many of the points above.

Software and code

Policy information about availability of computer code

Data collection	No data was collected.
Data analysis	MATLAB, DSI Studio and Graph Pad Prism were used to conduct the experiments reported in the paper.

For manuscripts utilizing custom algorithms or software that are central to the research but not yet described in published literature, software must be made available to editors/reviewers. We strongly encourage code deposition in a community repository (e.g. GitHub). See the Nature Research guidelines for submitting code & software for further information.

Data

Policy information about availability of data

All manuscripts must include a data availability statement. This statement should provide the following information, where applicable:

- Accession codes, unique identifiers, or web links for publicly available datasets
- A list of figures that have associated raw data
- A description of any restrictions on data availability

The diffusion MRI datasets used in this study are publicly available in the following repositories:

1. DSI Datasets from Hagmann et al (https://doi.org/10.1371/journal.pbio.0060159 and http://umcd.humanconnectomeproject.org/)
2. DSI MGH-USC HCP Consortium (https://db.humanconnectome.org)
3. HARDI Datasets WU-Minn HCP Consortium Lifespan (https://www.humanconnectome.org/study-hcp-lifespan-pilot)
4. ABIDE-II ASD Datasets (http://fcon_1000.projects.nitrc.org/indi/abide/abide_II.html)

Field-specific reporting

Please select the one below that is the best fit for your research. If you are not sure, read the appropriate sections before making your selection.

☒ Life sciences ☐ Behavioural & social sciences ☐ Ecological, evolutionary & environmental sciences

For a reference copy of the document with all sections, see nature.com/documents/nr-reporting-summary-flat.pdf

Life sciences study design

All studies must disclose on these points even when the disclosure is negative.

Sample size	Diffusion MRI datasets from public on-line repositories, as detailed in the manuscript, were downloaded and used.
Data exclusions	No data excluded.
Replication	We replicate results presented in Figure 3 from Alstott et al [11], to support the correctness of our analysis. No other replication experiments were made.
Randomization	Not relevant. Groups obtained from publicly available datasets.
Blinding	Not relevant. Groups obtained from publicly available datasets.

Reporting for specific materials, systems and methods

We require information from authors about some types of materials, experimental systems and methods used in many studies. Here, indicate whether each material, system or method listed is relevant to your study. If you are not sure if a list item applies to your research, read the appropriate section before selecting a response.

Materials & experimental systems

n/a	Involved in the study	
☒	☐	Antibodies
☒	☐	Eukaryotic cell lines
☒	☐	Palaeontology
☒	☐	Animals and other organisms
☐	☒	Human research participants
☒	☐	Clinical data

Methods

n/a	Involved in the study	
☒	☐	ChIP-seq
☒	☐	Flow cytometry
☐	☒	MRI-based neuroimaging

Human research participants

Policy information about studies involving human research participants

Population characteristics	All datasets used in the manuscript were collected from human research participants by other researcher groups, and are publicly available in on-line databases (See Data section above). However, our study did not involve the recruitment of human research participants. All population characteristics are described in the manuscript.
Recruitment	No recruitment needed.
Ethics oversight	Not needed. Anonymized data were obtained from publicly available databases (with their own ethics oversight approvals).

Note that full information on the approval of the study protocol must also be provided in the manuscript.

Magnetic resonance imaging

Experimental design

Design type	Diffusion MRI (Note that other modalities, e.g. fMRI, were collected in some of the studies from which the publicly available datasets were obtained, but are not used in our study).
Design specifications	Not relevant to diffusion MRI.
Behavioral performance measures	Not relevant to diffusion MRI.

Acquisition

Imaging type(s)	Diffusion MRI
Field strength	3T
Sequence & imaging parameters	Details provided in the Methods section, as well as relevant cited publications.
Area of acquisition	Brain

Diffusion MRI ☒ Used ☐ Not used

Parameters: Details provided in the Methods section, as well as relevant cited publications.

Preprocessing

Preprocessing software	Preprocessed data were used as obtained from the public databases listed in the Data section.
Normalization	No normalization was performed. Analyses were performed in diffusion data native space.
Normalization template	Data was not normalized.
Noise and artifact removal	Preprocessed data were used as obtained from the public databases listed in the Data section.
Volume censoring	No volume censoring was performed.

Statistical modeling & inference

Model type and settings	Not relevant to structural connectivity matrix analyses.
Effect(s) tested	Not relevant to structural connectivity matrix analyses.

Specify type of analysis: ☒ Whole brain ☐ ROI-based ☐ Both

Statistic type for inference (See Eklund et al. 2016)	Unpaired t tests were used to obtain two-sided p values, with homoscedasticity assumption, and assess differences between groups for each node (cortical area).
Correction	Family-wise error rate (correction for multiple comparisons) was controlled using the Holm-Sidak method with Alpha = 0:05.

Models & analysis

n/a	Involved in the study
☒	☐ Functional and/or effective connectivity
☐	☒ Graph analysis
☒	☐ Multivariate modeling or predictive analysis

Graph analysis	Connectivity matrices (graphs) were constructed with weights defined as the number of streamlines (from tractography) connecting each pair of cortical areas (per unit surface for Hagmann et al data). Nodal measures (e.g. cuvature) were computed from those matrices.
